# Upper Gastrointestinal Bleeding Due to Metastatic Lung Adenocarcinoma in the Stomach and Duodenum

**DOI:** 10.14309/crj.0000000000001474

**Published:** 2024-08-22

**Authors:** Taha Bin Arif, Bedoor Alabbas, Rakesh Vinayek

**Affiliations:** 1 Department of Internal Medicine, Sinai Hospital of Baltimore, Baltimore, MD; 2 Department of Gastroenterology and Hepatology, Sinai Hospital of Baltimore, Baltimore, MD

**Keywords:** lung adenocarcinoma, metastases, upper gastrointestinal bleeding, esophagogastroduodenoscopy, hemostatic clip

## Abstract

There are very few reports of bloodborne metastasis of lung adenocarcinoma to the gastrointestinal tract, primarily due to poor prognosis and short survival rate of metastasized carcinoma. We present a case of a 79-year-old man with a medical history of lung adenocarcinoma, who presented with complaints of weakness and melena for 1 week. He had symptomatic anemia, for which he was transfused with blood. Esophagogastroduodenoscopy showed a 10 mm sessile polyp in the gastric body that was removed. One month later, the patient presented with a similar complaint, and another esophagogastroduodenoscopy revealed 2 ulcerated lesions in the second portion of the duodenum. These lesions were treated by hemostatic clip placement and heater probe coagulation. Biopsy of lesions demonstrated thyroid transcription factor 1 and Napsin-positive tumor cells, consistent with lung adenocarcinoma. Owing to the poor prognosis of lung adenocarcinoma metastasizing to the lymph nodes, stomach, and duodenum, the patient was transferred to hospice care.

## INTRODUCTION

Blood-borne metastasis of the gastrointestinal (GI) tract is rare. The 3 most common tumors that metastasize to the GI tract are melanomas, breast cancers, and lung cancers.^1^ Although these extra-abdominal tumors can spread to any portion of the GI tract, the stomach and small intestine have been reported to be common sites.^2^ As patients with GI metastasis are usually asymptomatic, they are detected incidentally on imaging or autopsy. There are few reports of GI bleeding secondary to metastatic lung cancer in the literature. We present a case of symptomatic anemia due to upper GI bleeding caused by metastasis of primary lung adenocarcinoma to the stomach and duodenum.

## CASE REPORT

A 79-year-old man with a medical history of chronic obstructive pulmonary disease, pulmonary embolism, atrial fibrillation (on apixaban), hypertension (on metoprolol), and recently diagnosed with lung adenocarcinoma with lymph node metastasis has not started on any treatment and presented from the oncology clinic with complaints of weakness and melena for 1 week.

**Figure 1.**
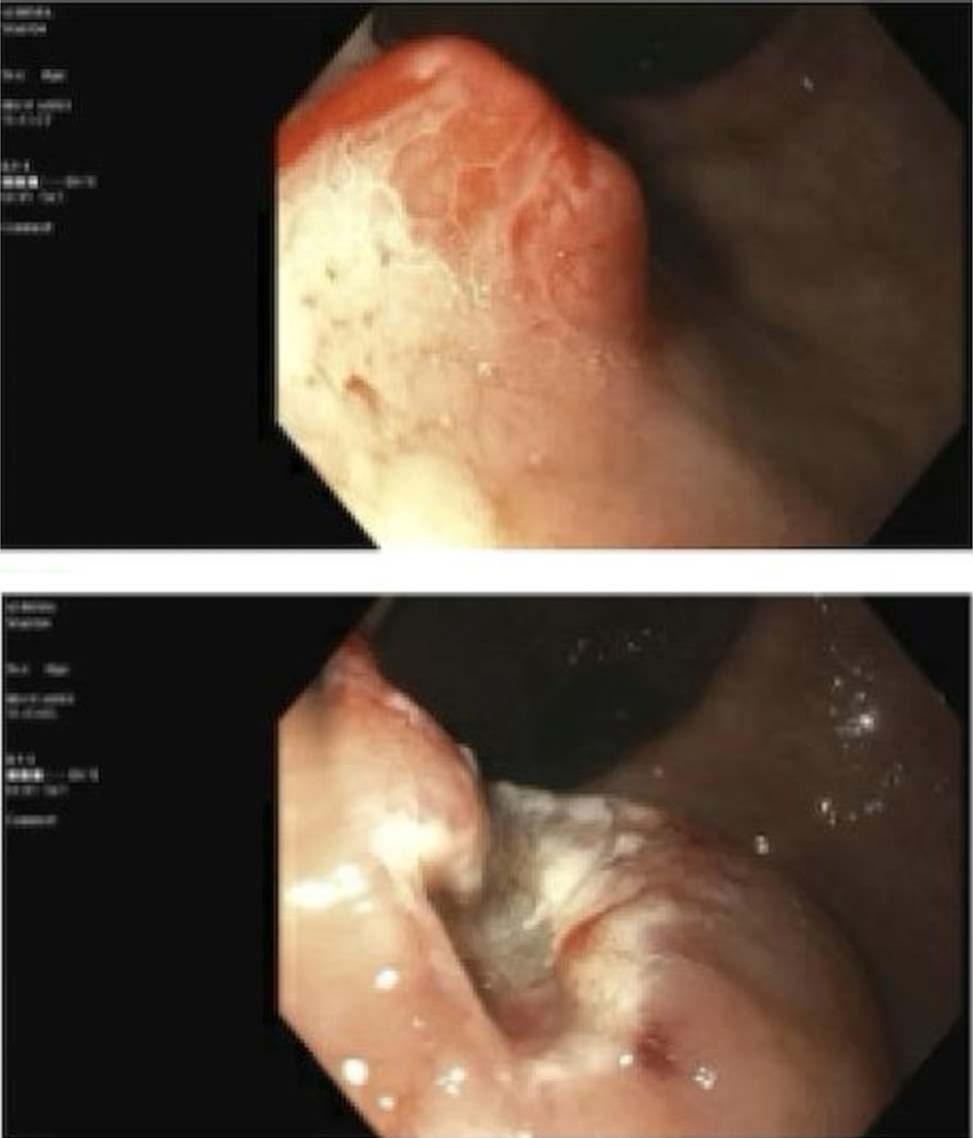
Gastric body polyp found on esophagogastroduodenoscopy.

On presentation, he was tachycardic, slightly hypotensive, and had an oxygen saturation of 97% on a 2 L nasal cannula. Initial laboratory results showed anemia with hemoglobin of 4.6 g/dL, hematocrit of 14.6%, and thrombocytopenia (104,000 per microliter). The coagulation profile was within the normal range. A recent iron panel was consistent with iron deficiency anemia. He was transfused with 2 units of packed red blood cells and was started on intravenous pantoprazole. Aspirin, metoprolol, and apixaban were held due to symptomatic anemia and hypotension. Computed tomography of the chest, abdomen, and pelvis with intravenous contrast showed spiculated thin 1.9 cm density in posterior right upper lobe; semiconfluent small nodules and elongated densities at posterior left lung base; right supraclavicular, mediastinal, hilar, and peripancreatic adenopathy likely secondary to metastases from lung cancer; numerous colonic diverticula; and enlarged prostate gland. He underwent an upper endoscopy that showed a 10 mm sessile polyp with stigmata of recent bleeding on the posterior gastric wall and duodenal erosion (Figure 1). The polyp was removed using the endoscopic mucosal resection technique, and the duodenal erosion was clipped. A clip was placed after the polypectomy to prevent postpolypectomy bleeding. The pathology of the gastric body polyp was consistent with poorly differentiated metastatic adenocarcinoma. The patient was discharged home with a plan to follow-up with oncology and the initiation of immune therapy.

**Figure 2.**
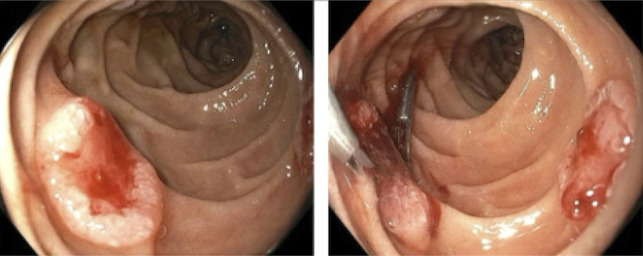
Bleeding ulcerated lesions in the second portion of duodenum found on esophagogastroduodenoscopy.

**Figure 3.**
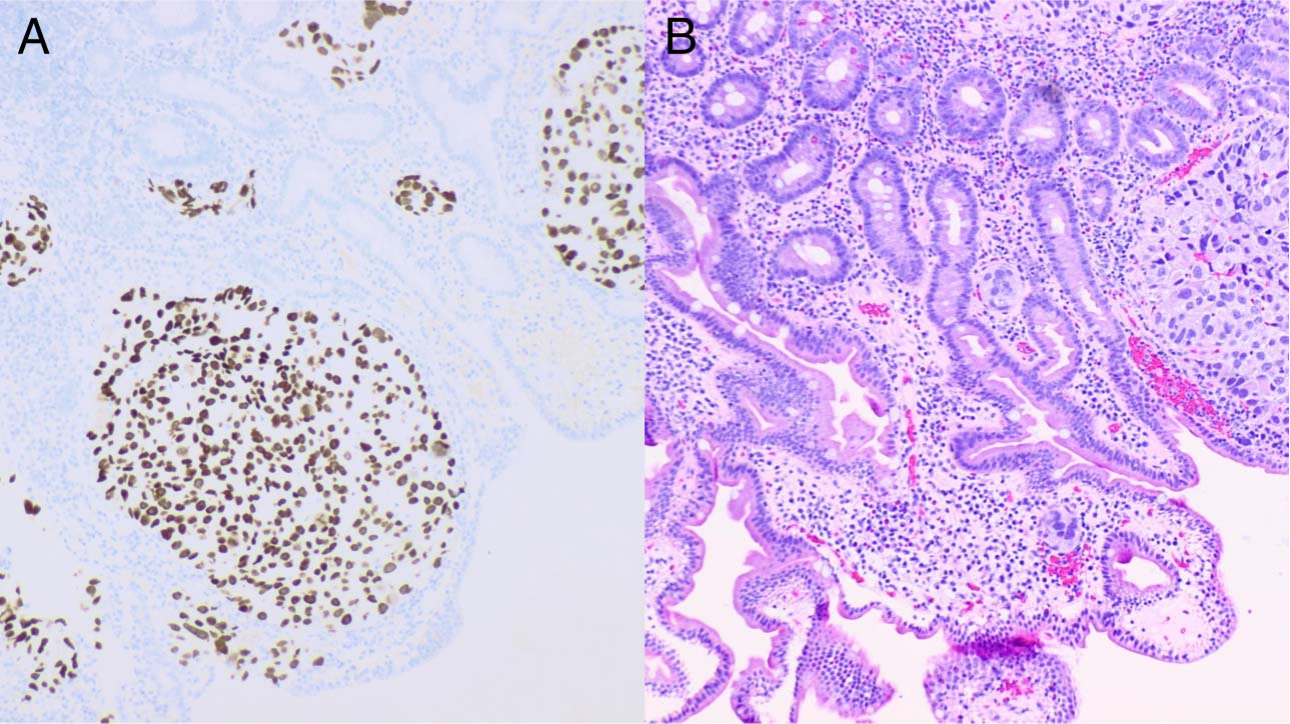
Histopathologic examination of ulcerated lesions of duodenum (A: Tumor cells positive for thyroid transcription factor 1, B: hematoxylin and eosin stain showing the clusters of tumor cells within the lamina propria including in lymphatic spaces). 100× magnification.

The patient was readmitted again one month after his initial presentation with a similar presentation including melena and anemia with a hemoglobin of 4.6. Esophagogastroduodenoscopy showed normal esophagus and previous clip in the gastric body at the previous polypectomy site without stigmata of recent bleeding. Two ulcerated lesions with oozing were found in the second portion of the duodenum (Figure 2). These lesions were treated by hemostatic clip placement and heater probe coagulation. Biopsies were taken from these duodenal sites. Surgical pathology of biopsy specimens revealed that duodenal lesions had tumor cells positive for thyroid transcription factor 1 (TTF-1) and focally positive for Napsin A, consistent with metastatic poorly differentiated adenocarcinoma from primary lung cancer (Figure 3). Owing to the poor prognosis, palliative care was involved after a discussion with medical oncology. The patient was discharged to home with home hospice.

## DISCUSSION

Metastases to the GI tract are rare in patients with primary lung cancer and are seen in the advanced stages of the disease. The reported incidence of symptomatic GI metastasis from primary lung cancer was 1.77% with abdominal pain and weight loss being the most observed symptoms.^3,4^ However, the prevalence at autopsy ranges from 4.7% to 14%.^5^ The probable factors leading to low incidence of metastasis from lung cancer are poor prognosis of such cases and short survival associated with metastatic lung cancer.^1,5^ Lung cancer metastasis to the small intestine often clinically presents with intestinal perforation and obstruction.^6,7^ Although the pathogenesis of perforation is not clearly understood, it is observed that these tumors have a greater tendency to undergo necrosis before attaining enough bulk. This has also led to a proposal that lung cancer should be considered a primary cancer if the metastatic tumor is unexpectedly found on the laparotomy for a perforated small bowel in a heavy smoker after the age of 50 years.^6^

There are few reports of GI metastasis from primary lung cancer presenting with GI bleeding as in our patient. The radiologic appearance of GI metastatic lesions depends on the histologic characteristics of the lesion including the degree of vascularity relative to the growth rate and the desmoplastic capability.^8^ Although there are very rare reports of radiologic features of metastatic lung cancer to the small bowel, a grossly necrotic small bowel mass with evidence of perforation and associated lymphadenopathy can be seen on computed tomography scans. About 34% of cases of lung cancer metastasis to the small bowel are through blood-borne metastasis. However, there have been reports of lung cancer spreading through direct invasion of the esophagus.

The most common histologic type of lung cancer metastasizing to the gut is squamous cell carcinoma followed by large cell carcinoma. Adenocarcinoma and small cell carcinoma have the lowest incidence compared with other types.^6,8^ Similarly, patients with small bowel metastasis from the lungs have at least another metastatic site, most commonly lymph nodes.^6^ Our patient had metastasis of lung adenocarcinoma spread to the supraclavicular, mediastinal, hilar, and peripancreatic lymph nodes; stomach; and the second part of the duodenum.

Lung cancer metastasis to the intestine does not exhibit any specific feature on endoscopy. It presents with diffuse involvement of intestinal mucosa, and multiple nodules with or without mucosal ulceration, or appears as solitary volcano-like tumors in the intestine.^9^ Pathological diagnosis using immunohistochemical staining is a reliable method to differentiate between primary small intestinal tumors and metastasis from the lungs.^10^ Primary lung cancer usually exhibits CK7+/CK20− immunophenotype while primary intestinal adenocarcinomas are CK7−/CK20+. In addition, lung adenocarcinoma has tumor cells positive for TTF-1 with a positive predictive value of >90%.^9,10^ In our case, the tumor cells were positive for TTF-1 and Napsin A, and their expressions were consistent with lung adenocarcinoma. Similarly, the previous pathology result of gastric polyp had tumor cells staining diffusely and strongly with CK7, Napsin, and TTF-1; patchy weak staining of CDX2; and negative for CK20.

In conclusion, our case highlights that patients with a history of lung adenocarcinoma presenting with GI bleeding and symptomatic anemia could have metastasis to multiple portions of the GI tract. Atypical symptoms can cause repeated hospitalizations and result into delay in immunotherapy, leading to overall poor prognosis. Physicians should be mindful of this rare association. Our case also highlights how fast the lung adenocarcinoma metastasizes to different portions of GI tract considering that duodenal lesions appeared with in 1 month of gastric metastasis. Optical diagnostic techniques can offer a promising noninvasive approach for improving the diagnosis of gastroduodenal lesions. We emphasize the potential of optical diagnosis to identify the histology of gastric polyps before resection, potentially avoiding unnecessary resections in cases of metastatic lesions. Collaboration between gastroenterologists and pathologists is essential for the optimal management of patients with gastric polyps and suspected metastatic lesions. Endoscopy with pathologic examination of biopsied specimens can detect such metastatic tumors earlier and provide hemostatic therapy.

## DISCLOSURES

Author contributions: TB Arif is the guarantor of the article who contributed to the conception of article and drafted the manuscript. B. Alabbas and R. Vinayek critically analyzed the manuscript and provided valuable feedback. All authors contributed towards the acquisition of data, approved the final version of the manuscript, and agree to be responsible for the accuracy and integrity of the study.

Financial disclosure: None to report.

Informed consent was obtained for this case report.
